# Sex- and Metamorphosis-Related Changes in the Cuticular Lipid Profile of *Galleria mellonella* Pupae and Adults

**DOI:** 10.3390/insects15120965

**Published:** 2024-12-04

**Authors:** Mieczysława I. Boguś, Michalina Kazek

**Affiliations:** 1 Museum and Institute of Zoology, Polish Academy of Sciences, Twarda 51/55, 00-818 Warsaw, Poland; 2 Biomibo, 15 Strzygłowska St., 04-872 Warsaw, Poland; 3 Centre for Advanced Materials and Technologies (CEZAMAT), Warsaw University of Technology, 19 Poleczki St., 02-822 Warsaw, Poland; michalina.kazek@pw.edu.pl

**Keywords:** *Galleria mellonella*, metamorphosis, copulation, cuticular free fatty acids, cholesterol, GC/MS

## Abstract

The majority of insects reproduce sexually. Among the many factors involved in controlling the reproductive process, cuticular lipids play an important role as unique chemical signatures of species, developmental stage, and sex, and participate in mate recognition. An understanding of the sex- and metamorphosis-related fluctuations in the cuticular lipid profiles of harmful insects is necessary to hamper their reproductive process. A GC/MS analysis of the cuticular lipids of the beehive pest *Galleria mellonella* Linnaeus (Lepidoptera: Pyralidae) revealed 11 FFAs in the male pupae (C8:0, C9:0, C14:0, C15:0, C16:1, C16:0, C17:0, C18:1, C18:0, C20:1, and C21:1) together with another two in the females (C10:0 and C17:1). As metamorphosis progressed, some FFAs disappeared from the pupal cuticle (C8:0 and C17:0 in both sexes, and C10:0, C17:1, and C20:1 only in female pupae) and the levels of the others changed. In adult virgin males and females, C8:0, C17:1, and C17:0 reappeared and two FFAs absent in pupae (C6:0 and C11:0) appeared. In virgin males, C13:0 also appeared (absent in pupae). Copulation resulted in the disappearance of C13:0 and C17:1, decreased the concentrations of C9:0, C11:0, C18:1, and C18:0, and elevated the amounts of C14:0, C16:1, and C16:0 in mated males. In mated females, the concentrations of C11:0, C14:0, C15:0, C16:0, C17:1, and C18:1 increased while C18:1 decreased. Copulation reduced cholesterol levels in mated females, and increased those in males.

## Simple Summary

Cuticular lipid composition is extensively studied in social insects. Our work is the first to investigate metamorphosis-related changes and the effect of copulation on the cuticular free fatty acid (FFA) profile of male and female nonsocial *Galleria mellonella* Linnaeus (Lepidoptera: Pyralidae). The radical reconstruction occurring inside the pupal body is accompanied by qualitative and quantitative changes in the cuticular FFA profiles on its surface, which differ between male and female pupae. A GC/MS analysis revealed the presence of 11 FFAs (from C8:0 to C21:1) in male pupae, and 13 FFAs in females. Adult virgin moths possess significantly fewer FFAs than pupae, but demonstrate three FFAs that are absent from pupae. Copulation significantly affected the composition of the FFAs and cholesterol concentration in the cuticle.

## 1. Introduction

Insects, the dominant form of life on Earth, have achieved evolutionary success thanks in part to efficient mechanisms that guide reproduction, a complex and fundamental biological process allowing the propagation of the species. The vast majority of insects reproduce sexually. After being fertilized by male sperm during copulation and insemination, the female lays yolky eggs [1,2,3,4]. The process of reproduction involves multiple stages and is influenced by the physiological condition of the individual insects; particularly important roles are played by their nutritional status, the degree of development of the gonads and maturity of the gametes, and external factors such as ambient temperature and humidity, day length, courtship conditions, copulation, and the availability of food for the offspring [2,5,6,7,8,9]. Also, to ensure that reproduction is triggered under optimal environmental and biological conditions, the reproductive system is under precise neurohormonal control that synchronizes environmental signals with the insect’s internal physiological state [1,10,11,12,13].

A particularly important role is played by the lipids covering the insect cuticle. Cuticular lipid profiles present unique chemical signatures determined by species, developmental stage, sex, and physiological state, and mediate various inter- and intra-species interactions, such as the regulation of mate recognition and courtship behavior [14,15,16,17,18,19]. Low-molecular volatile cuticular compounds act as pheromones that allow insects to find a partner for copulation even from a very large distance. Such pheromones are used in traps aimed at controlling harmful insects, and their production is a growing industry [20]. Targeting one or more key elements of the reproductive process can have a significant impact on the pest population [21]; hence, it is important to understand the cuticular lipid profiles of insect pests and the changes that occur in them during metamorphosis and are related to sexual maturation.

As bees play a distinct role in agriculture, there has been considerable scientific interest in controlling their pests and parasites. Among these is the greater wax moth, *Galleria mellonella* (Pyralidae): a widespread destructive pest of honeybee hives that causes severe damage to weakened or neglected hives and stored comb, especially in warmer areas [22]. Although its biology is well known, mainly because the larvae are used in research models replacing vertebrates in toxicological and immunological studies [23], the role of its cuticular lipids in the reproductive process remains unclear. The aim of this work is to determine the differences in cuticular lipid profiles between females and males, and confirm whether the dynamics of the changes taking place in these profiles during metamorphosis and sexual maturation proceed similarly in both sexes. Such studies are made possible by the clear sexual dimorphism of females and males in the pupal and imago stages [23].

## 2. Materials and Methods

### 2.1. Insects

Cultures of wax moths, *G. mellonella* (Pyralidae, Lepidoptera), were reared in glass chambers on a semi-artificial diet composed of wheat flour, wheat bran, dry milk, corn flour, dry yeast, glycerine, honey, and water, as described by Sehnal [24]; the insects were kept at 30 °C, 70% relative humidity, and in constant darkness. Freshly emerged (one-day-old; 1dP), three-day-old (3dP), and six-day-old pupae (6dP), and three-day-old (3dA) sexually mature adults (divided into two groups: virgin moths and moths after copulation) were used for lipid extraction. In each group, at least five generations were bred under these conditions. To avoid copulation, the pupae were divided into female and male groups and kept separately. The male pupa can be identified by the presence of a pair of small, rounded knobs representing the phallomeres, described by Kwadha [25], while the female pupae have a single copulatory aperture on segment IX [23,26]. The virgin males and females were paired for 24 h (10 males and 10 females in a separate container; the experiment was performed in five separate replicates, *n* = 10 per both sexes in each replicate) and then separated again. The insects selected for cuticular lipid extraction were weighed, frozen, and kept at −80 °C till the time of extraction.

### 2.2. Extraction of Cuticular Free Fatty Acids, Derivatization, and GC/MS Analyses

Cuticular lipid components containing FFAs were extracted and analyzed by gas chromatography coupled with mass spectrometry (GC/MS). The larvae were weighed and subjected to extraction for five minutes in 20 mL of dichloromethane (Merck; Darmstadt, Germany). The extracts were placed in glass flasks and evaporated using a nitrogen evaporator (XcelVap; Horizon Technology, St. Marys, PA, USA). Trimethylsilyl esters (TMSs) of FFAs were obtained by adding 100 μL of BSTFA:TMCS mixture 99:1 (Merck; Darmstadt, Germany) to 1 mg of each extract, and heating for one hour at 100 °C. The TMSs of the fatty acids were then analyzed by GC/MS. The analyses were carried out on a Shimadzu GCMS-QP2010 with a mass detector (Kyoto, Japan) and NIST 11 mass spectra database, as was described previously in detail by Kazek [27,28].

### 2.3. Statistics

All the statistical analyses were performed using Student’s *t*-test and one-way ANOVA, with the results being significant at *p* ≤ 0.05. The calculations were performed using STATISTICA 6.0 (StatSoft Polska; Kraków, Poland) and GraphPad 8.0 (La Jolla, CA, USA).

## 3. Results

### 3.1. Developmental Changes in the Cuticular Lipids Composition of G. mellonella Pupae

**Figure 1:**
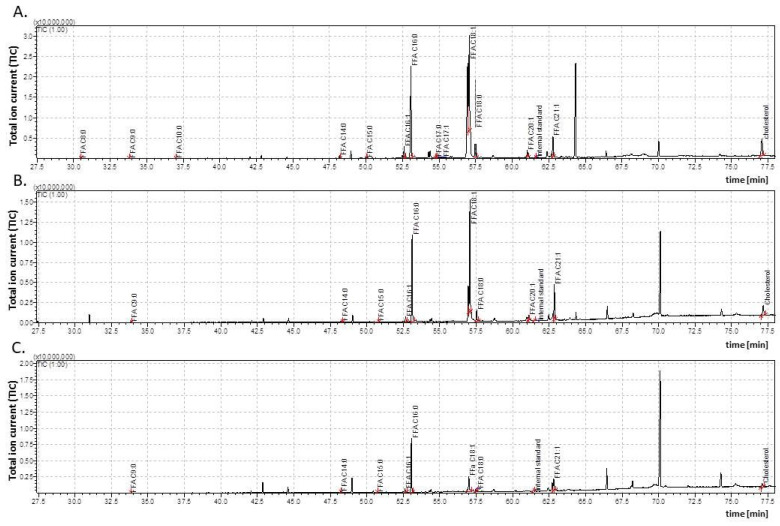
The total ion current (TIC) of fatty acids (TMSs) of the dichloromethane extract from the *Galleria mellonella* female pupae (**A**)—1-day-old (1dP-F); (**B**)—3-day-old (3dP-F); (**C**)—6-day-old (6dP-F). IS—internal standard (19-methylarachidic acid); fatty acids and molecular ions: octanoic acid (C8:0, *m*/*z* = 216), nonanoic acid (C9:0, *m*/*z* = 230), tetradecanoic acid (C14:0, *m*/*z* = 300), pentadecanoic acid (C15:0, *m*/*z* = 314), hexadecenoic acid (C16:1, *m*/*z* = 326), hexadecanoic acid (C16:0, *m*/*z* = 328), heptadecenoic acid (C17:1, *m*/*z* = 340), heptadecanoic acid (C17:0, *m*/*z* = 342), octadecenoic acid (C18:1, *m*/*z* = 354), octadecanoic acid (C18:0, *m*/*z* = 356), eicosenoic acid (C20:1, *m*/*z* = 382), and heneicosenoic acid (C21:1, *m*/*z* = 396). The mass spectra of the tested TMSs revealed the presence of the ions: M^+^ (molecular ion), [M-15]^+^, and fragment ions at *m*/*z* 117, 129, 132. The contents were calculated by comparing the relative peak areas with the IS peak area.

**Figure 2:**
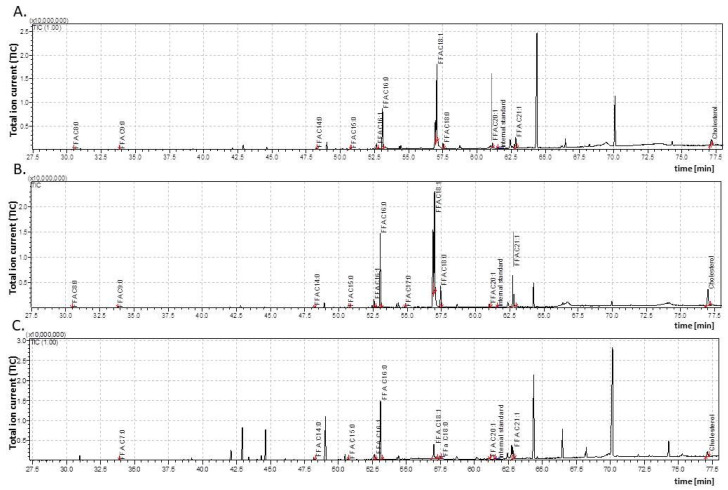
The total ion current (TIC) of fatty acids (TMSs) of the dichloromethane extract from the *Galleria mellonella* male pupae (**A**)—1-day-old (1dP-M); (**B**)—3-day-old (3dP-M); (**C**)—6-day-old (6dP-M). IS—internal standard (19-methylarachidic acid); fatty acids and molecular ions: octanoic acid (C8:0, *m*/*z* = 216), nonanoic acid (C9:0, *m*/*z* = 230), decanoic acid (C10:0, *m*/*z* = 244), tetradecanoic acid (C14:0, *m*/*z* = 300), pentadecanoic acid (C15:0, *m*/*z* = 314), hexadecenoic acid (C16:1, *m*/*z* = 326), hexadecanoic acid (C16:0, *m*/*z* = 328), heptadecenoic acid (C17:1, *m*/*z* = 340), heptadecanoic acid (C17:0, *m*/*z* = 342), octadecenoic acid (C18:1, *m*/*z* = 354), octadecanoic acid (C18:0, *m*/*z* = 356), eicosenoic acid (C20:1, *m*/*z* = 382), and 21-heneicosenoic acid (C21:1, *m*/*z* = 396). Further explanations are given in Figure 1.

Under optimal growth conditions, *G. mellonella* remains in the pupal stage for approximately one week. Both the male and female pupae were taken one, three, and six days after pupation (1dP, 3dP, and 6dP, respectively) for the GC/MS analysis of cuticular FFAs. The results were used to determine quantitative and qualitative differences in the cuticular FFA content (Figure 1 and Figure 2; Table 1).

The total mass of the FFAs extracted from the pupal cuticle significantly differed between males and females. The highest concentration of total FFAs was extracted from the male pupae at the beginning of the pupal stage (1dP-M; 90,491.28 ± 59,846.35 µg/g body mass), after which the concentration of the FFAs decreased 4.9-fold (3dP-M; 18,341.99 ± 3199.02 µg/g body mass), reaching a similar value to that of the one-day-old female pupae (1dP-F; 18,047 ± 6499.42 µg/g body mass). In turn, the female cuticle reached its highest total FFA content on the third day of the pupal stage (3dP-F; 50,471.82 ± 1904.06 µg/g body mass); following this, this value decreased 2.7-fold (6dP-F; 18,368.32 ± 1119.48 µg/g body mass). At the end of the pupal stage (6dP), no significant difference in the total FFA concentration was observed between females and males.

However, significant differences in the qualitative composition of cuticular FFAs were found between females and males. The cuticle of the male pupae contained 11 FFAs (C8:0, C9:0, C14:0, C15:0, C16:1, C16:0, C17:0, C18:1, C18:0, C20:1, and C21:1), while the female cuticle contained these, together with another two (C10:0 and C17:1). Interestingly, C10:0 and C17:1 were present only in the one-day-old female pupae (1dP-F) and disappeared during metamorphosis. Furthermore, C8:0 and C17:0 also disappeared in both the female and male pupae over time, while C20:1 was lost only from the six-day-old female pupae (6dP-F). The predominant FFAs in all the developmental pupal stages, in both sexes, were C16:1, C16:0, C18:1, and C18:0, while the lowest concentrations were noticed in the case of short-chain acids (C8:0, C9:0, and C10:0).

Our data indicates that while the concentrations of individual FFAs changed during the pupal stage in all the insects (Table 1), the dynamics of these changes differed between the sexes both with regard to the level and direction of changes (i.e., increases vs. decreases vs. stabilization). Particularly distinct differences in the course of these changes were noted for C9:0, C14:0, C18:0, and C21:1.

In addition to the FFAs, cholesterol was found in all the extracts obtained from the pupae (Table 2). The highest concentration of cholesterol was observed in the extract from the one-day-old male pupae (1dP-M; 6984.93 ± 389.71 μg/g insect body mass); this value fell 5.8-fold in 3dP-M (1206.50 ± 103.97 μg/g insect body mass) and then slightly increased in 6dP-M (1588.60 ± 615.21 μg/g insect body mass). In contrast, the female pupae demonstrated lower cholesterol concentrations: the 1dP-F value was 6.2 times lower than in males of the same age (1121.43 ± 615.21 μg/g insect body mass), but then rose 2.6-fold in 3dP-F before falling to the initial levels.

### 3.2. Cuticular Lipid Composition of the G. mellonella Adults

**Figure 3:**
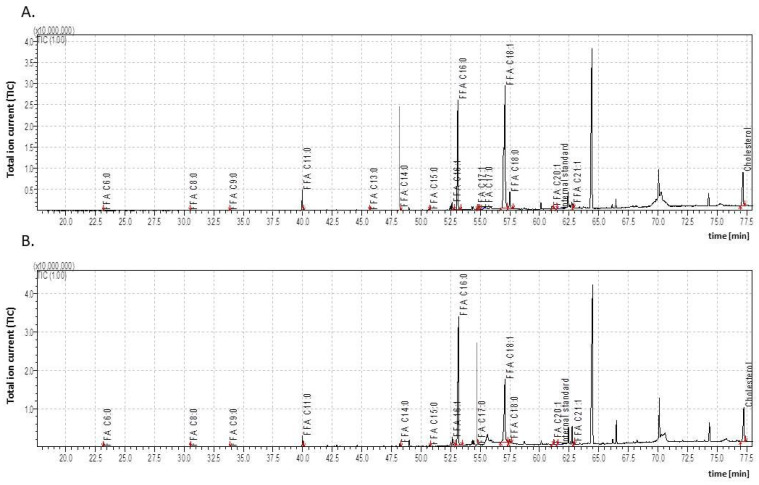
The total ion current (TIC) of fatty acids (TMSs) of the dichloromethane extract from the *Galleria mellonella* adults (**A**)—virgin male (3dA-MV); (**B**)—male after copulation (3dA-MC). IS—internal standard (19-methylarachidic acid); fatty acids and molecular ions: hexanoic acid (C6:0, *m*/*z* = 188), octanoic acid (C8:0, *m*/*z* = 216), nonanoic acid (C9:0, *m*/*z* = 230), undecanoic acid (C11:0, *m*/*z* = 258), tridecanoic acid (C13:0, *m*/*z* = 286), tetradecanoic acid (C14:0, *m*/*z* = 300), pentadecanoic acid (C15:0, *m*/*z* = 314), hexadecenoic acid (C16:1, *m*/*z* = 326), hexadecanoic acid (C16:0, *m*/*z* = 328), heptadecenoic acid (C17:1, *m*/*z* = 340), heptadecanoic acid (C17:0, *m*/*z* = 342), octadecenoic acid (C18:1, *m*/*z* = 354), octadecanoic acid (C18:0, *m*/*z* = 356), eicosenoic acid (C20:1, *m*/*z* = 382), heneicosenoic acid (C21:1, *m*/*z* = 396), tetracosanoic acid (C24:0, *m*/*z* = 440), and 23-hexacosanoic acid (C26:0, *m*/*z* = 468). Further explanations are given in Figure 1.

**Figure 4:**
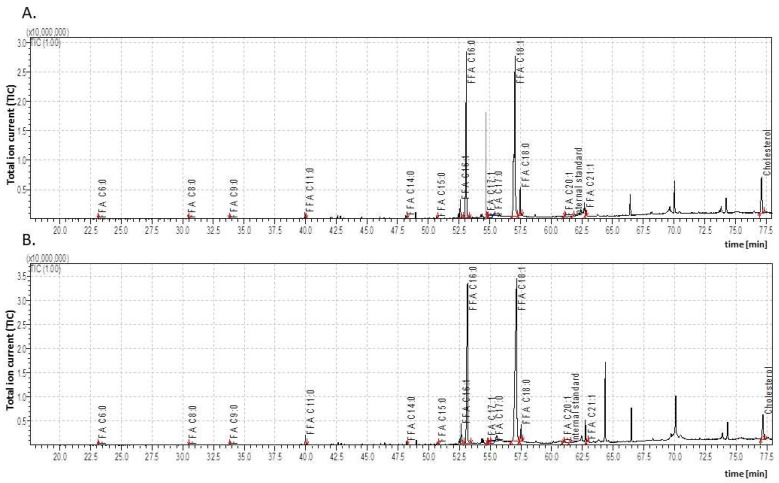
The total ion current (TIC) of fatty acids (TMSs) of the dichloromethane extract from the *Galleria mellonella* adults (**A**)—virgin female (3dA-FV); (**B**)—female after copulation (3dA-FC). IS—internal standard (19-methylarachidic acid); fatty acids and molecular ions: hexanoic acid (C6:0, *m*/*z* = 188), octanoic acid (C8:0, *m*/*z* = 216), nonanoic acid (C9:0, *m*/*z* = 230), undecanoic acid (C11:0, *m*/*z* = 258), tridecanoic acid (C13:0, *m*/*z* = 286), tetradecanoic acid (C14:0, *m*/*z* = 300), pentadecanoic acid (C15:0, *m*/*z* = 314), hexadecenoic acid (C16:1, *m*/*z* = 326), hexadecanoic acid (C16:0, *m*/*z* = 328), heptadecenoic acid (C17:1, *m*/*z* = 340), heptadecanoic acid (C17:0, *m*/*z* = 342), octadecenoic acid (C18:1, *m*/*z* = 354), octadecanoic acid (C18:0, *m*/*z* = 356), eicosenoic acid (C20:1, *m*/*z* = 382), heneicosenoic acid (C21:1, *m*/*z* = 396), tetracosanoic acid (C24:0, *m*/*z* = 440), and 23-hexacosanoic acid (C26:0, *m*/*z* = 468). Further explanations are given in Figure 1.

The GC/MS analysis indicated that the cuticular FFA content also differed both quantitatively and qualitatively between the adult males and females (Figure 3 and Figure 4; Table 3). In the virgin males (3dA-MV), the total cuticular FFA content was 8798.69 ± 482.44 µg/g of body mass, i.e., 1.8 times less than in pupae one day before adult eclosion (6dP-M; 16,142.46 ± 2733.48 µg/g of body mass; Table 1); they also presented 15 compounds from C6:0 to C21:1, of which four were absent in the male pupae (C6:0, C11:0, C13:0, and C17:1). Interestingly, while C8:0 and C17:0 were absent in 6dP-M and 3dP-M, respectively, both reappeared in the adult males. The virgin male cuticle (3dA-MV) also contained significantly lower concentrations of almost all the FFAs compared to the 6dP-M pupae, i.e., one day before eclosion; these values ranged from 1.5 to 18.9 times lower. The most substantial decrease in concentration occurred in the case of C21:1. The concentrations of C18:1 and C18:0 each increased by 1.5-fold.

The adult virgin females (3dA-FV) also demonstrated a similar pattern of changes, although the decrease in the total FFAs was slightly smaller (1.5 times) than in males. The total concentration of FFAs in the virgin females (12,155.02 ± 612.95 body mass) was 1.4 times higher than in the virgin males (8798.69 ± 482.44 µg/g body mass). In addition, the virgin females had one fewer FFA detected than males (C13:0 was absent); the concentrations of FFAs ranged from 1.5 to 21.5 times lower than in pupae (6dP-F), while C18:1 were elevated 1.8-fold and C18:0 1.1-fold.

In the adult virgin females (3dA-FV), the levels of all the individual FFAs differed significantly from those measured in the pupal cuticle (6dP-F). In the virgin males (3dA-MV), all the FFA levels were significantly different apart from C18:0 and C20:1 compared to pupae (6dP-M). In both the adult males and females, the same FFAs predominated as in the pupae (C16:0, C16:1, C18:0, and C18:1).

Cholesterol concentrations in pupae (6dP-M and 6dP-F) did not differ significantly from those in virgin adults, neither males (3dA-MV) nor females (3dA-FV). In turn, the cuticle cholesterol concentration in the virgin adult females (3dA-FV) was 1.2 times higher than in the virgin males (3dA-MV; Table 2).

### 3.3. The Impact of Copulation on Cuticular Lipid Composition of Adult G. mellonella

All the individuals studied copulated, although it was not possible to determine whether these were single or multiple copulations, or whether there was any exchange of partners. Copulation did not affect the total FFA concentration in males or females, but it should be noted that the cuticle of the adult females contained significantly higher total FFA content than the cuticle of males (Figure 3 and Figure 4; Table 3). Copulation, however, had a significant effect on FFA composition. In males, it caused the disappearance of C13:0 and C17:1; decreased the concentrations of C9:0, C11:0, C18:1, and C18:0; and increased those of C14:0, C16:1, and C16:0. In the females, C11:0, C14:0, C15:0, C16:0, C17:1, and C18:1 were elevated while C18:0 decreased.

Copulation had different effects on the cuticular cholesterol levels in adult males and females (Table 2). The cholesterol levels fell (1.6-fold) in the copulated females, and rose (1.4-fold) in males.

## 4. Discussion

Our analysis of the FFA composition in the cuticle of the pupae and adult moths of *G. mellonella* yielded very similar findings to those of a previous study on two-day-old pupae (2dP) and two-day-old adults (2dA), but without distinguishing between males and females [29]. The cuticular FFAs found to predominate in the *G. mellonella* pupae and adults (C16:0, C16:1, C18:0, and C18:1) were also found to be dominant in various other insects, even evolutionarily distant species [30,31,32,33,34,35,36,37,38]. This is not surprising, as FFAs containing 16 and 18 carbon atoms are the most widely distributed in nature [39].

Our previous studies on cockroaches (*Blatta orientalis* and *Blattella germanica*) and flies (*Calliphora vomitoria*, *Lucilia sericata*, and *Sarcophaga carnaria*) also indicate considerable changes in cuticular FFA profiles at various stages of development [33,34,35,36]; in addition, works on *Chorthippus brunneus*, *Dermester ater*, and *Dermestes maculatus* revealed differences in FFA profiles between adult males and females [37,38]. Our present findings demonstrate for the first time that the FFA composition of the *G. mellonella* pupal cuticle differs between males and females. While sex differences probably also occur during the life of the wax moth larva, the lack of sexual dimorphism in the larval stage [23] unfortunately makes it impossible to confirm this.

During the pupal stage, the insect undergoes a thorough hormonally controlled reconstruction involving complex changes in form and function. Larval structures are removed, mainly through autophagic and apoptotic cell death, and replaced with new adult structures, created based on imaginal disks containing quickly proliferating cells [23,40,41,42,43,44]. The radical changes that occur inside the wax moth pupa are accompanied by the cuticle changing from yellowish white just after pupation to dark brown before the adult emerges. The pupal cuticle also undergoes rapid sclerotization and the eyes become visible on the second day after pupation [23,25,26]. Our present findings demonstrate that these morphological changes are accompanied by changes in FFA profiles, and that the dynamics of these changes differ between males and females.

It is interesting that some FFAs disappear from the pupal cuticle with age and during metamorphosis, viz. C8:0 and C17:0 in both sexes but C10:0, C17:1, and C20:1 only in females, and the concentrations of the other FFAs change, but no new FFAs appear. This is not surprising considering how little is known about sex-specific changes in the FFA profiles associated with metamorphosis. It remains obscure why in both sexes, C8:0 and C17:0 disappear from the cuticle of pupae with age; similarly, it is not clear why C10:0 and C17:1 occur only in the cuticle of the female pupae, and only at the beginning of the pupal stage; why C17:0 only appears at the beginning of the pupal stage in both sexes; and why C20:1 disappears from female pupae the day before adult moth eclosion.

Most intriguingly, following successful metamorphosis, the FFAs C8:0, C17:0, and C17:1 reappear in the adult females and males, and these are accompanied by new FFAs which were absent in the pupal stage (C6:0, C11:0, and C13:0 in males only). Currently, the reasons for the reappearance of these fatty acids and their functions remain unclear; further research is needed to determine the functions of these compounds and their possible involvement in metamorphosis.

Our findings indicate that of all the detected cuticular lipids, only two FFAs: C13:0 (tridecanoic acid) and C17:1 (heptadecenoic acid), present in adult males, disappear after copulation. Tridecanoic acid is absent in both virgin and post-copulation females, as well as in the pupae of both sexes, suggesting it might play a male-specific role in reproduction, although there are currently no data available on what role this might be. Moreover, while many species of insect possess androconia, i.e., scent scales involved in pheromone production in males, they have not been identified in *G. mellonella*; as such, it is difficult to associate this fatty acid with any male-specific structure [45,46]. Tridecanoic acid has been found in the bodies of the wheat stink bug *Aelia rostrata*, black soldier fly *Hermetia illucens*, mealworm *Tenebrio molitor*, and on the cuticle of the larvae, pupae, and adults of fly *Sarcophaga argyrostoma*, but the functions performed by this fatty acid in these insect species remain obscure [47,48,49,50,51]. Tridecanoic acid is used as a food-flavoring agent, antibacterial, and antibiofilm agent; it also inhibits the survival of *Escherichia coli* and biofilm formation, and can be used in bacterial infection research [52,53,54].

In turn, C17:1 (heptadecenoic acid) disappears from the cuticle of the adult males after copulation, but is present in both the virgin and post-copulation females; interestingly, in the mated females, the level of C17:1 increases in a statistically significant way, which may suggest its transfer from the male to the female cuticle during mating. However, it is not known whether this transfer may be active or passive and what function C17:1 may perform in adult moths because little is known about the biological functions of this fatty acid. Heptadecenoic acid (C17:1), a minor constituent of ruminant fats with an unclear origin [55], is noticeably antagonistic towards the growth of the oral bacterium *Streptococcus sobrinus* [56].

While the increase in the concentration of C11:0, C17:1, and C18:1 in the mated females after copulation is associated with a decrease in males, suggesting their transfer between cuticles, the increase in the concentration of C14:0, C15:0, and C16:0 cannot be explained in this way, as the levels also increase in the male. However, the fact that the concentration of C18:0 falls in both females and males may indicate its consumption during copulation, or a similar mechanism of its distribution in the body, in both sexes.

In many insect species, copulation and the transfer of sperm and other components of the ejaculate trigger the reproductive processes of the females, the final effect being the laying of fertilized eggs. The changes in gene expression induced by mating in female insects are currently the subject of intensive study, with particular emphasis on the genes involved in metabolism control, immunity, and chemosensory [57]. Variable gene expression levels have been measured in *G. mellonella* larvae during the course of bacterial infection [58], while a group of 57 genes, some of them sex-specific, involved in chemoreception and mainly expressed in antennae were identified in adults using high-throughput sequencing [59].

It should be noted that *G. mellonella* displays a unique form of mating behavior in which the male emits short pulses of sound at a frequency of 75 kHz, which attracts virgin females [23,60]. The acoustic signal is generated using structures found on the wings [61,62]. Females react to the sound signal by fanning their wings [63]. When a male and a female come close to each other, the male releases a mixture of short-range sex pheromones, including nonanal; decanal; hexanal; heptanal; undecanal; 6,10,14-trimethylpentacanol-2; and 5,11-dimethylpentacose, which initiate mating; among these, only nonanal and undecanal were identified as behaviorally active [64,65,66,67,68,69]. Nonanal is a saturated fatty aldehyde arising from the reduction of the carboxyl group of nonanoic acid (C9:0); it is found in numerous essential oils and shows high potential in seizure control [70,71]. At present, it is difficult to predict whether the significant reduction in nonanoic acid (C9:0) concentration in the male cuticle after copulation (Table 3) is related to nonanal production. The situation is even less clear-cut in the case of undecanal, a saturated fatty aldehyde arising from the reduction of the carboxyl acid group of undecanoic acid (C11:0). It is known to have antifungal effects which are believed to stem from its effect on the long-chain acyl-coenzyme A synthetase I [72,73,74]. Undecanoic acid (C11:0) is present in the cuticle of both male and female wax moths, both virgin and mated; however, its concentration significantly decreases in males after copulation and increases in mated females. The metabolic pathways for converting undecanoic acid to undecanal and vice versa are not known, so it is difficult to clearly link the observed changes in the concentration of C11:0 with the production and release of undecanal by male *G. mellonella.*

The influence of copulation on cuticular lipid composition has been extensively studied in social insects, where chemical communication plays a key role in determining social roles, and cuticular lipid profiles encode information on queen age, fertility, and mating status, which could be utilized by workers and rival queens [75,76,77]. Our findings are the first to present the effect of copulation on the cuticular free fatty acid profile of nonsocial insects.

A summary of the fatty acids studied, their potential biological functions, and occurrences in *G. mellonella* is presented in Table 4.

While cholesterol is fundamental for cell function, acting as a membrane constituent that regulates their fluidity, insects lack the essential enzymes needed to synthesize cholesterol de novo, and are, therefore, dependent on the presence of cholesterol in the diet [102,103,104]. Studies on dietary cholesterol absorption and tissue distribution in *Rhodnius prolixus* indicate that only a small amount of cholesterol absorbed by the midgut was found in the hemolymph, where it was observed in the free form or associated with lipophorin, but mainly accumulated by the fat body [104]. Although no data exists on the transport of cholesterol to the insect cuticle, it cannot be ruled out that this may be supported by apolipoproteins, which are known to deliver hydrocarbons to the cuticle [105]. Further studies are needed to explain the metamorphosis-related differences in cholesterol concentration occurring in the pupal stage in both sexes, as well as the changes following copulation in adult moths.

## Figures and Tables

**Table 1:** Content of free fatty acids present on the pupae cuticle of *Galleria mellonella* during metamorphosis.

FFA	*Galleria mellonella* Male Pupae [µg/g of Body Mass ± SD]			*Galleria mellonella* Female Pupae [µg/g of Body Mass ± SD]		
	1-Day-Old (1dP-M)	3-Days-Old (3dP-M)	6-Days-Old (6dP-M)	1-Day-Old (1dP-F)	3-Days-Old (3dP-F)	6-Days-Old (6dP-F)
C8:0	68.78 ± 20.07 ^A,1^	21.39 ± 8.80 ^A^	ND	10.94 ± 2.37 ^1^	ND	ND
C9:0	66.67 ± 18.06 ^B,2^	56.64 ± 11.32 ^C,3^	20.47 ± 2.01 ^B,C,4^	15.29 ± 2.72 ^a,2^	92.58 ± 16.83 ^a,3^	42.09 ± 6.48 ^a,4^
C10:0	ND	ND	ND	6.52 ± 0.88	ND	ND
C14:0	435.38 ± 51.47 ^D,5^	134.77 ± 8.32 ^D,6^	369.64 ± 45.13 ^7^	96.28 ± 7.16 ^b,c,5^	231.49 ± 21.97 ^b,6^	224.86 ± 8.39 ^c,7^
C15:0	140.24 ± 30.22 ^E,8^	42.02 ± 3.98 ^E,F,9^	108.87 ± 29.71 ^F^	16.39 ± 1.61 ^d,e,8^	107.01 ± 36.75 ^d,9^	99.55 ± 19.08 ^e^
C16:1	1486.49 ± 936.72	600.32 ± 117.38 ^10^	907.44 ± 182.26	366.38 ± 24.68 ^f^	951.28 ± 14.43 ^f,10^	746.47 ± 31.79 ^f^
C16:0	16,150.93 ± 12,860.83	5506.82 ± 537.76 ^11^	8977.88 ± 1003.65	4252.08 ± 405.22 ^g^	16,944.05 ± 286.35 ^g,11^	10,506.37 ± 118.53 ^g^
C17:1	ND	ND	ND	25.85 ± 4.47	ND	ND
C17:0	7520.28 ± 12,729.01	ND	ND	31.50 ± 2.20	ND	ND
C18:1	47,279.24 ± 31,126.33	9353.28 ± 1688.69 ^G^	3355.67 ± 712.66 ^G^	11,160.84 ± 5769.14 ^h^	22,588.88 ± 768.59 ^h,i^	3766.79 ± 870.94 ^i^
C18:0	5346.14 ± 435.76 ^H^	599.47 ± 122.99 ^H,12^	265.53 ± 78.37 ^H^	1186.17 ± 73.48 ^j^	1955.70 ± 111.08 ^j,12^	465.15 ± 3.47 ^j^
C20:1	1380.79 ± 358.62 ^I,13^	758.48 ± 232.45	400.62 ± 226.18 ^I^	245.07 ± 39.14 ^13^	560.29 ± 231.69	ND
C21:1	10,616.36 ± 1279.24 ^J,K,14^	1268.81 ± 467.32 ^J,15^	1736.35 ± 453.50 ^K,16^	634.26 ± 166.33 ^k,14^	7040.53 ± 416.33 ^k,15^	2517.03 ± 60.79 ^k,16^
Total	90,491.28 ± 59,846.35 ^L,M,17^	18,341.99 ± 3199.02 ^L,18^	16,142.46 ± 2733.48 ^M^	18,047.59 ± 6499.42 ^l,17^	50,471.82 ± 1904.06 ^l,m,18^	18,368.32 ± 1119.48 ^m^

Statistically significant differences are marked with the same letter or number: inside the male groups with capital letters (^A–M^), and small letters (^a–m^) were used for the female pupae. Differences between males and females of the same age are marked with numbers (^1–18^). ANOVA, Tukey test, and *t*-test, *p* < 0.05; SD—standard deviation; FFA—free fatty acid; ND—not detected.

**Table 2:** Content of cholesterol present on the cuticle of *Galleria mellonella* pupae and adults.

*Galleria mellonella* Male Pupae [µg/g of Body Mass ± SD]			*Galleria mellonella* Female Pupae [µg/g of Body Mass ± SD]			*Galleria mellonella* Male Adults [µg/g of Body Mass ± SD]		*Galleria mellonella* Female Adults [µg/g of Body Mass ± SD]	
1-Day-Old (1dP-M)	3-Day-Old (3dP-M)	6-Day-Old (6dP-M)	1-Day-Old (1dP-F)	3-Day-Old (3dP-F)	6-Day-Old (6dP-F)	Virgin (3dA-MV)	After Copulation (3dA-MC)	Virgin (3dA-FV)	After Copulation (3dA-FC)
6984.93 ± 389.71 ^A,!^	1206.50 ± 103.97 ^#^	1588.60 ± 615.21 ^A^	1121.43 ± 35.30 ^a,!^	2969.90 ± 186.91 ^a,b,#^	1193.59 ± 79.97 ^b^	1084.47 ± 24.76 ^&,$^	1492.96 ± 20.87 ^&,@^	1306.33 ± 73.95 ^%,$^	826.04 ± 19.27 ^%,@^

Statistically significant differences between the pupae of the same sex but different ages are marked with the same letters: male pupae (^A^) and female pupae (^a,b^). Special signs are used to mark significant differences between the male and female pupae in the same age: 1dP-M vs. 1dP-F (^!^), 3dP-M vs. 3dP-F (^#^) and between virgin adults: 3dA-MV vs. 3dA-FV (^$^), mated adults: 3dA-MC vs. 3dA-FC (^@^) and effect of copulation in male (^&^; 3dA-MV vs. 3dA-MC) and female (^%^; 3dA-FV vs. 3dA-FC) adults. ANOVA, Tukey test, and *t*-test, *p* < 0.05; SD—standard deviation.

**Table 3:** Content of free fatty acids present on the cuticle of *Galleria mellonella* adults and the effect of copulation.

FFA	*Galleria mellonella* Male Adults [µg/g of Body Mass ± SD]		*Galleria mellonella* Female Adults [µg/g of Body Mass ± SD]	
	Virgin (3dA-MV)	After Copulation (3dA-MC)	Virgin (3dA-FV)	After Copulation (3dA-FC)
C6:0	2.16 ± 0.34 ^A^	1.82 ± 0.37	3.16 ± 0.30 ^A^	2.28 ± 1.00
C8:0	3.49 ± 0.68	3.50 ± 0.24	4.13 ± 1.44	2.83 ± 1.35
C9:0	8.14 ± 0.59 ^B,1^	5.15 ± 1.26 ^1^	5.20 ± 0.82 ^B^	5.44 ± 0.47
C11:0	323.64 ± 6.57 ^C,2^	227.09 ± 39.64 ^2^	112.08 ± 11.08 ^C,8^	171.37 ± 5.27 ^8^
C13:0	5.43 ± 1.98	ND	ND	ND
C14:0	47.83 ± 2.38 ^3^	69.77 ± 10.95 ^3^	47.27 ± 2.64 ^9^	59.79 ± 2.14 ^9^
C15:0	17.73 ± 0.73 ^D^	24.24 ± 10.78	23.87 ± 2.49 ^D,10^	31.79 ± 0.17 ^10^
C16:1	121.79 ± 15.09 ^E,4^	203.20 ± 43.44 ^a,4^	330.97 ± 15.67 ^E^	351.39 ± 12.01 ^a^
C16:0	2641.69 ± 73.17 ^F,5^	4875.65 ± 442.90 ^5^	4236.01 ± 198.71 ^F,11^	4831.52 ± 6.64 ^11^
C17:1	20.98 ± 2.33	ND	20.23 ± 2.41 ^12^	29.81 ± 3.07 ^12^
C17:0	17.69 ± 2.93 ^G^	22.27 ± 11.17	32.38 ± 2.09 ^G^	44.21 ± 9.50
C18:1	5009.43 ± 274.66 ^H,6^	2661.65 ± 695.75 ^b,6^	6672.04 ± 300.57 ^H,13^	7392.67 ± 224.19 ^b,13^
C18:0	405.94 ± 79.49 ^7^	119.57 ± 49.32 ^c,7^	537.13 ± 22.96 ^14^	319.57 ± 57.16 ^c,14^
C20:1	81.29 ± 12.55 ^I^	41.66 ± 31.86	13.79 ± 2.87 ^I^	49.16 ± 12.97
C21:1	91.45 ± 8.92	57.41 ± 19.99	116.76 ± 48.88	52.84 ± 3.48
Total	8798.69 ± 482.44 ^J^	8312.99 ± 1357.67	12,155.02 ± 612.95 ^J^	13,344.69 ± 339.44

Statistically significant differences between virgin males and females are marked with the same capital letter (^A–J^), mated females and males are marked with the same small letter (^a–c^), virgin males and mated males are marked with the same digit (^1–7^), and virgin and mated females are marked with the same digit (^8–14^). ANOVA, Tukey test, and *t*-test, *p* < 0.05; SD–standard deviation; FFA—free fatty acid; ND—not detected.

**Table 4:** Summary of studied fatty acids, their functions, and occurrence in *Galleria mellonella*.

Fatty Acid (Common Name)	Chemical Formula	Occurrence in Developmental Stages	Occurrence after Copulation	Potential Biological Function(s)	References
Hexanoic acid (Caproic acid) C6:0	C_6_H_12_O_2_	Absent in pupae; present in adults	Present in both sexes	Intermediate in lipid metabolism; pheromone precursor; cell signaling	[78,79,80,81,82]
Octanoic acid (Caprylic acid) C8:0	C_8_H_16_O_2_	Disappears in pupae; reappears in adults	No change	Antimicrobial properties; cell signaling	[34,35,78,79,83]
Nonanoic acid (Pelargonic acid) C9:0	C_9_H_18_O_2_	Present in pupae and adults	Decreases in males post-copulation	Precursor of nonanal; likely role in pheromone production; antifungal properties; cell signaling	[64,65,66,67,68,69,70,71,78,79,84]
Decanoic acid (Caprinic acid) C10:0	C_10_H_20_O_2_	Present in female pupae only	Absent in adults	Potential cuticular barrier function; likely role in pheromone production; cell signaling; antifungal properties	[78,79,85]
Undecanoic acid (Hendecanoic acid) C11:0	C_11_H_22_O_2_	Absent in pupae; appears in adults	Increases in females; decreases in males post-copulation	Antifungal properties; potential conversion to undecanal in pheromone signaling	[34,35,86,87]
Tridecanoic acid (Tridecylic acid) C13:0	C_13_H_26_O_2_	Absent in pupae; appears in adult males only	Disappears post-copulation in males	Antimicrobial properties; male-specific reproductive role (hypothetical); likely role in pheromone production	[52,53,54,88]
Tetradecanoic acid (Myrystic acid) C14:0	C_14_H_28_O_2_	Present across all stages	Increases in both sexes post-copulation	Structural lipid; likely involved in energy metabolism; likely role in pheromone production	[89,90]
Pentadecanoic acid (Pentadecylic acid) C15:0	C_15_H_30_O_2_	Present across all stages	Increases in both sexes post-copulation	Antibacterial and structural functions; likely role in pheromone production	[91,92]
Hexadecenoic acid (Palmitoleic acid) C16:1	C_16_H_30_O_2_	Predominant in all stages	Increases in males post-copulation	Essential for cuticular water loss regulation; likely role in pheromone production; antifungal properties	[79,93,94]
Hexadecanoic acid (Palmitic acid) C16:0	C_16_H_32_O_2_	Predominant in all stages	Increases in both sexes post-copulation	Energy storage; cuticular rigidity; likely role in pheromone production; antifungal properties	[78,95,96]
Heptadecenoic acid C17:1	C_17_H_32_O_2_	Present in female pupae only	Increases in females; decreases in males post-copulation	Potential transfer during mating; unknown signaling role; antibacterial properties	[56]
Heptadecanoic acid (Margaric acid) C17:0	C_17_H_34_O_2_	Disappears in pupae; reappears in adults	No change post-copulation	Unknown; minor constituent in lipid profiles; likely role in pheromone production	[97]
Octadecenoic acid C18:1	C_18_H_34_O_2_	Predominant in all stages	Decreases in males; increases in females post-copulation	Structural and energy lipid; pheromone precursor; antifungal properties	[79,98,99]
Octadecanoic acid (Stearic acid) C18:0	C_18_H_36_O_2_	Predominant in all stages	Decreases in both sexes post-copulation	Likely metabolized during copulation; energy lipid; likely role in pheromone production; antifungal properties	[79,100]
Eicosenoic acid (Gondoic acid) C20:1	C_20_H_38_O_2_	Present in pupae; disappears in females before adulthood	Not detected in adults	Unknown; structural lipid function; antifungal properties	[79]
Heneicosenoic acid C21:1	C_21_H_40_O_2_	Present in all stages	Fluctuates in adults post-copulation	Cuticular hydrophobicity regulator; likely role in pheromone production	[27,101]

## Data Availability

The original contributions presented in the study are included in the article; further inquiries can be directed to the corresponding author/s.
